# Annealing temperature effect on self-assembled Au droplets on Si (111)

**DOI:** 10.1186/1556-276X-8-525

**Published:** 2013-12-13

**Authors:** Mao Sui, Ming-Yu Li, Eun-Soo Kim, Jihoon Lee

**Affiliations:** 1 College of Electronics and Information, Kwangwoon University, Nowon-gu, Seoul 139-701, South Korea; 2 Institute of Nanoscale Science and Engineering, University of Arkansas, Fayetteville, AR 72701, USA

**Keywords:** Au droplets, Annealing temperature, Nanowires

## Abstract

We investigate the effect of annealing temperature on self-assembled Au droplets on Si (111). The annealing temperature is systematically varied while fixing other growth parameters such as deposition amount and annealing duration clearly to observe the annealing temperature effect. Self-assembled Au droplets are fabricated by annealing from 50°C to 850°C with 2-nm Au deposition for 30 s. With increased annealing temperatures, Au droplets show gradually increased height and diameter while the density of droplets progressively decreases. Self-assembled Au droplets with fine uniformity can be fabricated between 550°C and 800°C. While Au droplets become much larger with increased deposition amount, the extended annealing duration only mildly affects droplet size and density. The results are systematically analyzed with cross-sectional line profiles, Fourier filter transform power spectra, height histogram, surface area ratio, and size and density plots. This study can provide an aid point for the fabrication of nanowires on Si (111).

## Background

Recently, nanoscale particles have drawn increasing attention. For example, gold particles, as a popular nanomaterial with outstanding optoelectronic properties, have been widely used in sensor applications by the enrichment of detection range and optimization and enhancement of sensitivity [1-4]. In addition, Au particles are also attractive based on their capacity to catalyze one-dimensional (1-D) nanostructures, namely nanopillars and nanowires with lots of remarkable properties via various epitaxial growth mechanisms [5-10]. Fabrications of diverse nanowires such as GaN, ZnO, InAs, GaAs, Si, and Ge have been demonstrated using Au droplets as catalyst [11-18]. Nonetheless, given the wide range of substrates utilized, Au droplets can be successfully utilized in the fabrication of the various nanowires and many elements utilized for substrates would diffuse into gold during the fabrications of nanowires [11-18]. The design and growth of nanowires including diameter, length, and density in many cases can be determined by the size, density, and configurations of Au droplets [17,18]. From this point, the control of Au droplet is an essential step for designing desired nanowires [19-24]. As discussed, the properties of Au droplets and approaches to the fabrication of nanowires have been widely studied; however, up to date, the systematic study on the control of Au droplets is still rarely to be studied.

**Figure 1:**
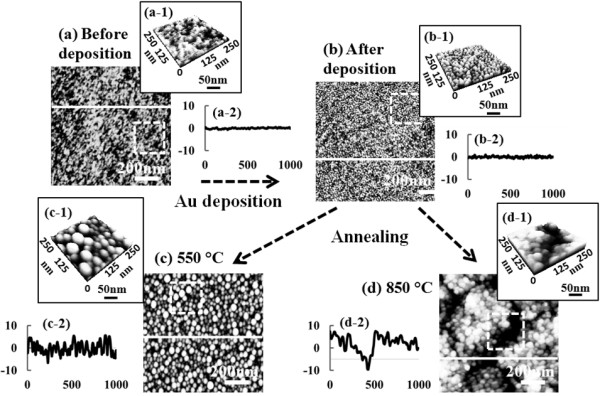
**Illustration of self-assembled Au droplet fabrication process on Si (111). (a)** shows AFM images of bare Si (111) and **(b)** shows the morphologies after 2-nm Au deposition before annealing. **(c)** and **(d)** present the surface morphologies of samples annealed at 550°C and 850°C, respectively. AFM top views in **(a)** to **(d)** are 1 × 1 μm^2^ and AFM side views of insets (a-1) to (d-1) are 250 × 250 nm^2^.

**Figure 2:**
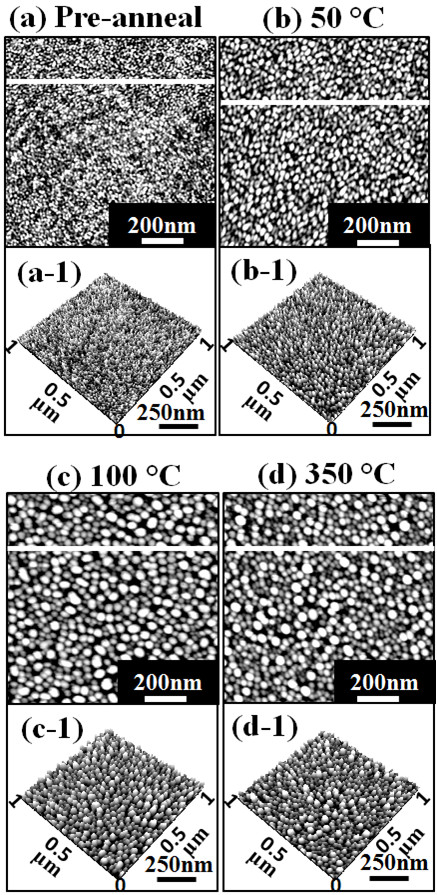
**Evolution of self-assembled Au droplets induced by variation of annealing temperature: from 50°C to 350°C.** Au droplets are fabricated with a fixed deposition amount of 2 nm and a fixed annealing duration of 30 s at each annealing temperature. **(a)** to **(d)** are AFM top views and (a-1) to (d-1) show AFM side views of 1 × 1 μm^2^.

In this paper, therefore, we investigate the annealing temperature effect of self-assembled Au droplets by systematically varying the annealing temperature on Si (111). To clearly observe the annealing temperature effect, the deposition amount and annealing duration are set to be fixed during the fabrication. For example, Figure 1 illustrates the general fabrication process of self-assembled Au droplets: bare Si (111) before the gold deposition in Figure 1(a) and after the Au deposition in Figure 1(b). Surfaces are quite very smooth before and even after 2-nm gold deposition as shown with surface line profiles in Figure 1(a-2) and (b-2). After deposition of 2-nm Au, the annealing temperature is systematically varied from 50°C to 850°C with a fixed Au deposition amount of 2 nm and a fixed annealing duration of 30 s. As examples, the resulting Au droplets at 550°C are shown in Figure 1(c) and at 850°C in Figure 1(d). After annealing at 550°C, self-assembled dome-shaped Au droplets are witnessed as clearly shown in Figure 1(c-1). However, the surface becomes quite segmented and coarse when the annealing temperature is reached to 850°C as shown in Figure 1(d-1).

## Methods

### Experimental details

In this work, gold droplets were synthesized on Si (111) substrates by the systematic variation of annealing temperature in a pulsed laser deposition (PLD) system under a chamber vacuum of 1 × 10^−4^ Torr. To investigate the annealing temperature effect on the fabrication of self-assembled Au droplets, each growth was performed at 50°C, 100°C, 150°C, 250°C, 350°C, 550°C, 700°C, 800°C, 850°C, 900°C, and 950°C, respectively. Initially, 1-mm-thick singular 4-in. p-type Si (111) wafers were 1 × 1 cm^2^ diced by a wire-sawing machine and treated with a conventional RCA clean. Each sample is degassed at 850°C for 15 min under a chamber vacuum of 1 × 10^−4^ Torr, and subsequently, 2-nm-thick gold films were deposited in a plasma ion-coater chamber under a pressure of 1 × 10^−1^ Torr at a rate of 0.05 nm/s with 3-mA ionization current. For a systematic annealing, a computer-operated recipe was run and the ramping-up rate was at 2.3°C/s under 1 × 10^−4^ Torr. After reaching each target annealing temperature, 30 s of annealing time was given for each sample, and finally, the temperature was quenched down immediately after finishing each growth to minimize Ostwald ripening [19,25]. The quenching process was kept identical for all samples. An atomic force microscope (AFM) was utilized for the surface morphology characterization, and XEI software was used for analyzing the obtained data.

## Results and discussion

**Figure 3:**
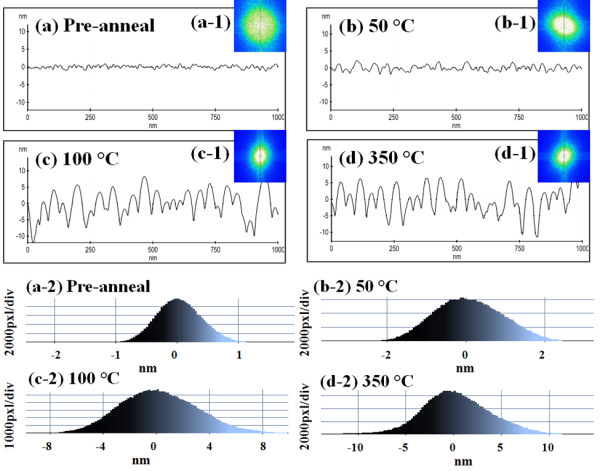
**Cross-sectional surface line profiles, 2-D FFT power spectra, and height distribution histograms around zero. (a)** to **(d)** show the cross-sectional surface line profiles, acquired from Figure 2 as indicated with white lines. Insets (a-1) to (d-1) are 2-D FFT power spectra and height distribution histograms around zero are shown in (a-2) to (d-2).

**Figure 4:**
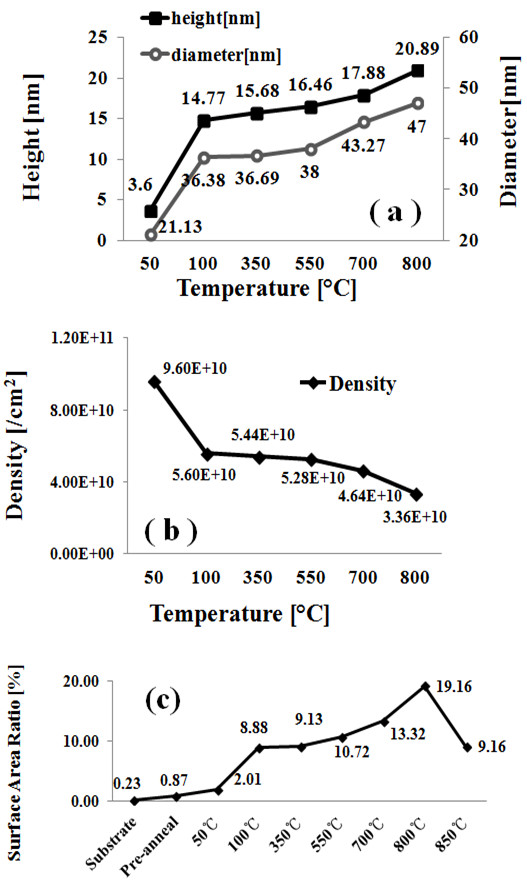
**Plots of AH, LD, AD, and surface area ratios of each sample. (a)** Plot summarizing the AH and LD of resulting self-assembled Au droplets at each annealing temperature. **(b)** Plot showing the AD of Au droplets. **(c)** Plot showing the surface area ratios of each sample, defined as [(Surface area − Geometric area)/Geometric area] × 100 (%).

Figure 2 shows the evolution of self-assembled Au droplets annealed between 50°C and 350°C on Si (111) with 2-nm-thick gold for 30 s. AFM top views are shown in Figure 2(a) to (d) and AFM side views are presented in Figure 2(a-1) to (d-1). Figures 3(a) to 4(d) show the cross-sectional surface line profiles acquired from the AFM images in Figure 2, which are indicated with white lines. The insets of Fourier filter transform (FFT) power spectra in Figure 3(a-1) to (d-1) represent the height information, converted from the spatial domain to the frequency domain by Fourier transform. Figure 3(a-2) to (d-2) are the height distribution histograms of each sample, which depict the height distribution around zero with Gaussian distribution. Figure 4a summarizes the average height (AH) and the lateral diameter (LD) of Au droplets versus the annealing temperature, and Figure 4b shows the average density (AD) of self-assembled Au droplets. Figure 4c shows the surface area ratios of corresponding samples at each condition. The surface area ratio is defined as the percentage of roughness of the surface given by [(Geometric area − Surface area) / (Geometric area)] × 100 (%). The surface area indicates three-dimensional (3-D) surface topology (*x* × *y* × *z*), and the geometric area is in 2-D (*x* × *y*). In general, the average size including the height and diameter of self-assembled Au droplets was gradually increased with correspondingly increased annealing temperature while the density of Au droplets was gradually decreased as clearly seen with the AFM images in Figure 2, the surface line profiles in Figure 3, and the plots of dimensions and densities in Figure 4a,b. For example, Figure 2(a) shows the Si (111) surface after 2-nm Au deposition, and the surface was very smooth as clearly seen with the line profile in Figure 3(a). The height distribution histogram (HDH) in Figure 3(a-2) shows ±1 nm. By annealing this sample at 50°C for 30 s, the nucleation of Au droplets with relatively smaller size was observed as seen in Figure 2(b) and (b-1). The AH of droplets at 50°C was 3.6 nm, the LD was 21.1 nm, and the AD was 9.6 × 10^10^/cm^2^ as shown in Figure 4a,b. The HDH became slightly wider to approximately ±2 nm in Figure 3(b-2). At 100°C, the size of droplets grew much larger and the density was reduced as shown in Figures 2(c) and 4. The AH of Au droplets was drastically raised by × 4.1 reaching 14.8 nm and the LD jumped by × 1.72 to approximately 36.4 nm. Meanwhile, the AD was dropped by × 1.71 to 5.6 × 10^10^/cm^2^ as compared to that at 50°C. Now, the HDH became much wider with the increased size of Au droplets to approximately ±8 nm in Figure 3(c-2). At 350°C, the droplets show a smaller increase in size and the density kept decreasing. The AH of Au droplets was 15.68 nm, the LD was 36.7 nm, and the AD was down to 5.44 × 10^10^/cm^2^ at 350°C. The HDH also showed a wider distribution with approximately ±10 nm in Figure 3(d-2). Along with the gradual size increase of self-assembled Au droplets by increased annealing temperatures, the surface area ratio (SAR) in Figure 4c also showed a progressively increasing trend. For example, the SAR was 0.23% for the bare and 0.87% for the pre-annealed sample, indicating very flat surfaces. Then, with the nucleation of mini Au droplets at 50°C, the SAR was raised to 2.01%. Then, the SAR jumped to 8.88% by over four times when the AH and LD of Au droplets were jumped at 100°C as seen in Figure 4c. Subsequently, as the Au droplet dimension was only slightly increased at 350°C, the SAR moderately increased to 9.13%. As another way of determining the surface roughness, the root-mean-squared (RMS) surface roughness (*R*_q_) of samples at corresponding annealing temperatures is summarized in Table 1. The *R*_q_ value reflects the direct change of surface morphology. The *R*_q_ was 0.376 nm for the pre-annealed surface after 2-nm gold deposition and slightly increased to 0.872 nm with the nucleation of droplets after annealing at 50°C. Then, it jumped to 3.701 nm at 100°C due to the formation of larger Au droplets as discussed and only slightly increased to 3.898 nm at 350°C. In terms of the shape uniformity, the surface before annealing with 2-nm gold deposition was quite flat and uniform as revealed in Figure 3(a), and thus, a very symmetric round FFT spectrum appeared as clearly shown in Figure 3(a-1). In the FFT power spectrum, the horizontal and vertical directions are given by taking the reciprocal of according units of horizontal and vertical directions in AFM images, and thus, the distribution of height is presented in distribution of colors with directionality. That is to say, symmetry of color distribution can reflect shape uniformity of Au droplets. With the nucleation of self-assembled Au droplets by annealing at 50°C, the FFT spectrum with a slight elongation along 135° and 315° was observed in Figure 3(b-1). The FFT power spectra at 100°C and 350°C also showed slight elongations in Figure 3(c-1) and (d-1). As mentioned, the distorted FFT power spectrum can be caused by lateral uniformity of nanostructures, and this could have been caused by the unfavorable Au adatom diffusion due to insufficient thermal energy at relatively lower annealing temperatures.

**Table 1:** **RMS surface roughness (*R***_**q**_**) of self-assembled Au droplets at corresponding annealing temperature**

	**Temperature (°C)**							
	**Pre-anneal**	**50**	**100**	**350**	**550**	**700**	**800**	**850**
*R*_q_ (nm)	0.376	0.872	3.701	3.898	4.024	4.158	6.856	3.912

*R*_q_ is gradually increased at 800°C and dropped at 850°C with droplet melting potentially due to the lower eutectic melting point.

**Figure 5:**
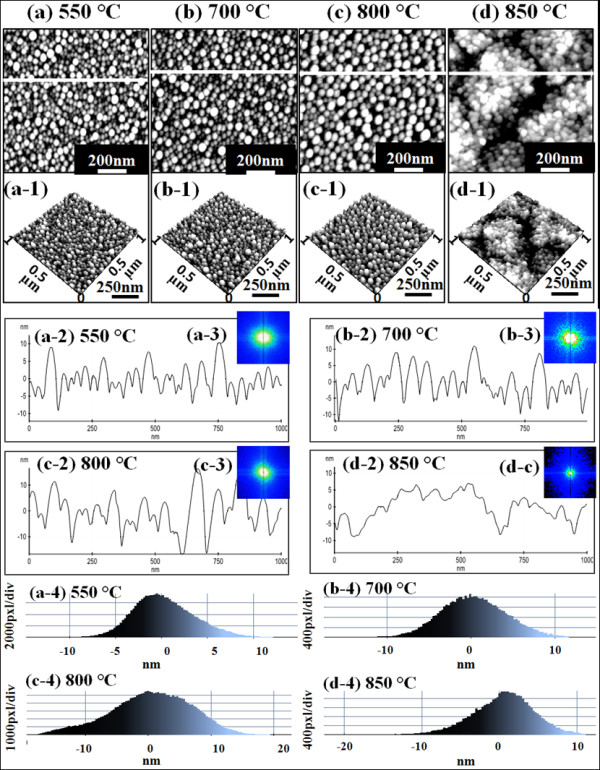
**Annealing temperature variation between 550°C to 850°C with 2-nm Au deposition for 30 s. (a)** to **(d)** are AFM top views and (a-1) to (d-1) show AFM side views of 1 × 1 μm^2^ areas. (a-2) to (d-2) show the cross-sectional surface line profiles, (a-3) to (d-3) are the 2-D FFT power spectra, and (a-4) to (d-4) are the height distribution histograms.

Figure 5 summarizes the resulting self-assembled Au droplets by annealing between 550°C and 800°C with 2-nm Au deposition and for 30 s of annealing at each growth temperature. In general, the size of droplets showed a gradual increase, and correspondingly, the density of droplets kept decreasing as seen in Figure 4a,b. For example, the AH and LD of Au droplets were approximately 16.6 and 38 nm, respectively, and the AD was 5.28 × 10^10^/cm^2^ at 550°C. The HDH was approximately ±10 nm in Figure 5(a-4). At 700°C, as shown in Figure 5(b), Au droplets slightly got larger and lower in density: the AH became approximately 17.9 nm, the LD was approximately 43.3 nm, and the AD was dropped to 4.64 × 10^10^/cm^2^. The HDH also got slightly wider to approximately ±11 nm in Figure 5(b-4). At 800°C, the size of droplets kept growing taller and larger, and inversely, the density got lower as summarized in Figure 4a,b: AH of approximately 20.9 nm, LD of 47 nm, and AD of 4.64 × 10^10^/cm^2^. The HDH now got much wider to approximately ±17 nm in Figure 5(c-4) perhaps due to the higher temperature. Finally, at 850°C, segmented rougher surface topology was observed in Figure 5(d) and (d-1), and the height of droplets became much smaller by melting as clearly seen with the line profile in Figure 5(d-2). The melting of Au droplets can be due to the lower eutectic point of Au-Si alloy. The eutectic point of Au-Si alloy can be determined by the concentration of Au/Si ratio [18,26-29], and a higher temperature can further accelerate Au and Si atom diffusion at the interface. Thus, the eutectic point of Au-Si alloy can be much lower than either Au or Si melting point. Although we did not add the data here, above 850°C (including 900°C and 950°C), the surface melting became even more severe and very ugly rough surface morphology resulted and the HDH showed bimodal distributions. The dimensional information at 850°C is omitted in the plots of Figure 4a,b. In terms of the SAR between 550°C and 800°C, with the size increase of droplets, the SAR also gradually increased: 10.72% at 550°C, 13.32% at 700°C, and 19.16% at 800°C. However, at 850°C, with the melting of Au droplets, the SAR was dropped to 9.16%. Similarly, the *R*_q_ between 550°C and 800°C kept increasing: 4.024 nm at 550°C, 4.158 nm at 700°C, and 6.856 nm at 800°C. Then, with the surface melting, the *R*_q_ got much reduced to 3.912 nm at 850°C, which is comparable to the one at 350°C. FFT power spectra of samples between 550°C and 800°C showed improved uniformities as shown in Figure 5(a-3) and (c-3) with symmetric round patterns as compared with the samples at 50°C to 350°C. With increased annealing temperature, the surface diffusion can become more favorable and thus better uniformity can result. At 850°C, the FFT got dimmer likely due to the melting. In short, as the annealing temperature was increased, the average density gradually decreased and the decrease in density was compensated by expansion of dimension, i.e., AH and LD. This trend, increased droplet dimensions associated with decreased density along with increased fabrication temperature, is a conventional behavior of metal droplets [30-32] and even of quantum structures and nanostructures [33-35] on various semiconductor surfaces. With increased annealing temperature, the surface diffusion as well as the diffusion length can be further enhanced, which consequently can result in increased dimension of metal droplets. The density can be higher at a lower temperature due to a shorter diffusion length with lower thermal energy and vice versa. Once droplets grow larger, they have lower surface energy and thus can attract more nearby adatoms and tend to grow larger until reaching the equilibrium. In any case, in general, the density change is accompanied with dimensional compensation.

**Figure 6:**
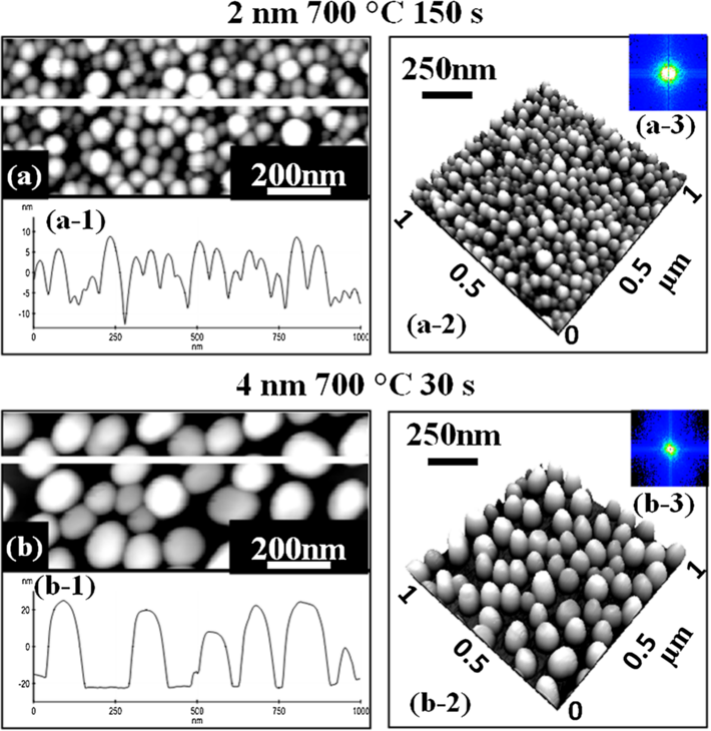
**Extended annealing duration and increased deposition amount effects and AFM side views. (a)** Extended annealing duration effect on self-assembled Au droplets. **(b)** Increased deposition amount effect. Au droplets in **(a)** are fabricated with 2 nm of Au deposition at 700°C with × 5 longer annealing duration of 150 s. In **(b)**, the Au droplets are fabricated with 30 s at 700°C with an increased deposition amount of 4 nm. **(a)** and **(b)** are AFM top views of 1 (*x*) × 0.5 (*y*) μm^2^ and (a-2) and (b-2) show AFM side views of 1 × 1 μm^2^.

Figure 6 shows Au droplets fabricated at an extended annealing duration in Figure 6(a) and with an increased deposition amount in Figure 6(b). Au droplets were fabricated at × 5 extended annealing duration of 150 s with the identical amount of 2 nm at 700°C, comparable with Figure 5(b). As shown with the AFM top view in Figure 6(a) and the side view in Figure 6(a-2), the resulting droplets are quite similar to those of the sample in Figure 5(b). For example, the size and density were quite similar and the uniformity was also similar, indicating that the extended annealing duration has a minor effect on the Au droplets. Figure 6(b) shows the self-assembled Au droplets with an increased deposition amount of 4 nm. With twice increased deposition amount, the Au droplets grew much bigger and taller and the density was significantly reduced. For example, the AH was approximately 48 nm and the LD was approximately 130 nm, which are approximately × 2.7 increased AH and approximately × 3 increased LD. The AD was 6.8 × 10^9^ cm^−2^ on average, which is approximately × 6.8 decrease as compared to the sample in Figure 5(b). It follows that while the increased annealing duration has a minor effect on the droplet size and density, the deposition amount can significantly affect the size and density of resulting droplets. Further studies are now underway for a more systematic study on deposition amount and annealing duration effects on self-assembled Au droplets.

## Conclusions

In brief, the annealing temperature effect on the fabrication of self-assembled Au droplets on Si (111) was studied in terms of size, density, and uniformity with AFM images, line profiles, FFT power spectra, and histograms. In general, the dimensions of Au droplets including the average height and diameter were gradually increased with the increased annealing temperature. The expansion of dimensions was accompanied by the reduction in the average density. The Au droplets fabricated below 500°C showed somewhat poor uniformities as evidenced by the FFT spectra, and the uniformity was improved between 550°C and 800°C likely due to favorable surface diffusion of adatoms induced by sufficient thermal energy. At above 850°C, the Au droplets began melting due to the lower eutectic point of Au-Si alloy, and the melting got severe as temperature was increased. With an increased deposition amount, the size of Au droplets grew much larger and the density was significantly decreased. Meanwhile, the increased annealing duration showed minor effects on the droplet size and density. This study can find applications in the fabrication of nanowires on Si (111).

## Competing interests

The authors declare that they have no competing interests.

## Authors' contributions

MS, ML, and JL participated in the experiment design and carried out the experiments. MS, ML, EK, and JL participated in the analysis of data. MS, ML, and JL designed the experiments and testing methods. MS and JL carried out the writing. All authors helped in drafting and read and approved the final manuscript.
